# Inflammatory Bowel Disease Is not Linked to a Higher Rate of Adverse Events in Colonoscopy—a Nationwide Population-based Study in Sweden

**DOI:** 10.1093/ecco-jcc/jjad114

**Published:** 2023-07-04

**Authors:** Bjarki T Alexandersson, Anna Andreasson, Charlotte Hedin, Gabriella Broms, Peter T Schmidt, Anna Forsberg

**Affiliations:** Karolinska Institutet, Department of Medicine Solna, Stockholm, Sweden; Karolinska Institutet, Department of Medicine Solna, Stockholm, Sweden; Stress Research Institute, Department of Psychology, Stockholm University, Stockholm, Sweden; School of Psychological Sciences Macquarie University, North Ryde, NSW 2109, Australia; Karolinska Institutet, Department of Medicine Solna, Stockholm, Sweden; Karolinska University Hospital, Department of Gastroenterology, Dermatovenerology and Rheumatology, Stockholm, Sweden; Gastroenterology, Danderyd hospital, Stockholm, Sweden; Division of Clinical Epidemiology, Department of Medicine Solna, Karolinska Institutet, Stockholm, Sweden; Department of Medical Sciences, Uppsala University, Uppsala, Sweden; Division of Clinical Epidemiology, Department of Medicine Solna, Karolinska Institutet, Stockholm, Sweden

**Keywords:** Gastrointestinal bleeding, perforation, inflammatory bowel disease

## Abstract

**Background and Aims:**

Inflammatory bowel disease may cause long-standing inflammation and fibrosis and may increase the risk of adverse events in colonoscopy. We evaluated whether inflammatory bowel disease and other potential risk factors are associated with bleeding or perforation in a nationwide, population-based, Swedish study.

**Methods:**

Data from 969 532 colonoscopies, including 164 012 [17%] on inflammatory bowel disease patients, between 2003 and 2019, were retrieved from the National Patient Registers. ICD-10 codes for bleeding [T810] and perforation [T812] within 30 days of the colonoscopy were recorded. Multivariable logistic regression was used to test if inflammatory bowel disease status, inpatient setting, time period, general anaesthesia, age, sex, endoscopic procedures, and antithrombotic treatment were associated with higher odds for bleeding and perforation.

**Results:**

Bleeding and perforation were reported in 0.19% and 0.11% of all colonoscopies, respectively. Bleeding [odds ratio 0.66, *p* <0.001] and perforation [odds ratio 0.79, *p* <0.033] were less likely in colonoscopies in individuals with inflammatory bowel disease status. Bleeding and perforation were more common in inpatient than in outpatient inflammatory bowel disease colonoscopies. The odds for bleeding but not perforation increased between 2003 to 2019. General anaesthesia was associated with double the odds for perforation.

**Conclusions:**

Individuals with inflammatory bowel disease did not have more adverse events compared with individuals without inflammatory bowel disease status. However, the inpatient setting was associated with more adverse events, particularly in inflammatory bowel disease status. General anaesthesia was associated with a greater risk of perforation.

## 1. Introduction

Colonoscopy is the current procedure of choice for the investigation of the lower gastrointestinal tract. It is generally a low-risk procedure, but adverse events occur. Population-based studies have shown incidence rates of post-colonoscopy bleeding across all indications to be between 0.05% and 1.1%, and incidence rates of post-colonoscopy perforations between 0.03% and 0.11%.^1–3^ A Swedish register study of colonoscopies between 2001 and 2013 reported an incidence rate of 0.17% for bleeding and 0.11% for perforation.^4^

Inflammatory bowel disease [IBD] has an incidence rate of 29/100 000 person-years in Sweden and is associated with significant morbidity and costs for society^5–7^ Colonoscopy is frequently performed in patients with IBD for diagnostic purposes and to evaluate the presence and severity of inflammation. Furthermore, due to the increased risk of colorectal cancer [CRC] in long-standing IBD, guidelines recommend regular surveillance colonoscopies^8,9^ Chronic inflammation of the intestines may lead to fibrosis, stenosis, and fistula,^10^ potentially increasing the risk of adverse events associated with colonoscopy in patients with IBD. However, previous studies have shown divergent results, with two studies showing an increase in the rate of perforation in IBD patients and two showing no difference compared with patients without IBD.^11–14^ However, only one of these studies was population-based and none of them was nationwide. No study has investigated if disease activity may influence the risk of adverse events in colonoscopy in patients with IBD.

The present, nationwide, population-based study aims to investigate the incidence of post-colonoscopy bleeding and perforation in Sweden from 2003 to 2019, and to determine if bleeding and perforation are more common in patients with IBD compared with patients without IBD, particularly in IBD patients with active disease. How the risk has changed over time, and the influence of risk factors including inpatient setting, general anaesthesia, procedures during colonoscopy and antithrombotic use, were also analysed.

## 2. Materials and Methods

### 2.1. Patients

This is nationwide, retrospective, cohort study of prospectively collected register data from the Swedish national patient registers and the Swedish prescribed drug register. These registers have previously been described.^4^ They cover almost 100% of the population and have been validated.^15^ By using personal identification numbers, data on each individual could be linked between the different registers. Information was collected on all individuals over 18 years of age, who had a colonoscopy between 2003 and 2019, by searching for the procedure codes for colonoscopy [UJF32 or UJF35]. The underlying population for this study was the entire adult population of Sweden in 2019 [about 8.3 million]. The study was approved by the Regional Ethics Review Board in Stockholm [Dnr 2015/690-31/2]. Informed consent was waived because of the retrospective nature of the study with the analysis using anonymised clinical data.

### 2.2. Outcome of adverse events

For this study we chose diagnostic codes that are specific for bleeding or perforation after a procedure [including colonoscopy], to avoid falsely capturing post-colonoscopy adverse events that had bleeding or perforation for other reasons. Bleeding or gastrointestinal haemorrhage was defined by the ICD-10 code T81.0 ‘Hemorrhage and hematoma complicating a procedure’ up to and including 30 days after the colonoscopy. Perforation was defined by the code T81.2 ‘Accidental puncture or laceration during a procedure’ up to and including 30 days after the colonoscopy.

### 2.3. Explanatory variables

#### 2.3.1. IBD status

IBD diagnosis was defined using the ICD-10 codes for Crohn’s disease [CD] K50.X, ulcerative colitis [UC] K51.X, and indeterminate colitis [IC] K52.3. We searched the Swedish inpatient and outpatient registers from and including 1987 to 2019. Individuals were classified as having IBD if they had two diagnoses of IBD before or up to 6 months after the colonoscopy.^16^ For patients with a diagnosis of both CD and UC, the most recent diagnosis at the time of colonoscopy was used. Where an individual had received a diagnosis of IC plus CD or UC, they were defined as having CD or UC, respectively. Individuals consistently assigned a diagnosis of IC on two or more occasions were defined as having IC. Disease duration was calculated from the first entry into the patient registries which included an IBD diagnosis to the time of the colonoscopy. Disease duration is shown as a descriptive variable in Table 1.

**Table 1.** Patient and procedure characteristics per colonoscopy, in total, and according to presence/absence of IBD and as well as separated on IBD subtypes.

	Total	Non-IBD	IBD	UC	CD	IC
	*N* = 969 532	*N* = 805 520	*N *= 164 012	*N* = 96 849	*N *= 64 027	*N* = 3136
Inpatient setting, *n* [%]	110,078 [11.4]	95,362 [11.8]	14,716 [9.0]	7,056 [7.3]	7.171 [11.2]	489 [15.6]
General anaesthesia, *n* [%]	17,969 [1.9]	12,257 [1.5]	5,712 [3.5]	2,286 [2.4%]	3,283 [5.1%]	143 [4.6%]
Women, *n* [%]	519,187 [53.6]	443,998 [55.1]	75,189 [45.8]	41,440 [50.1]	32,101 [52.6]	1,648 [52.6]
Age, mean [SE]	60 [17]	62 [16]	48 [17]	50[17]	47[17]	49[18]
Biopsy, *n* [%]	480,420 [49.6]	347,697 [43.2]	132,723 [80.9]	83,939 [86.7]	46,153 [72.1]	2,631 [83.9]
Polypectomy, *n* [%]	126,438 [13.0]	119,963 [14.9]	6,475 [3.9]	4,343 [4.5%]	2,001 [3.1%]	131 [4.2%]
Dilatation, *n* [%]	3,467 [0.4]	450 [0.1]	3,017 [1.8]	57 [0.1%]	2,953 [4.6%]	7 [0.2%]
Antithrombotic use, *n* [%]	176,393 [18.2]	163,523 [20.3]	12,870 [7.8]	8,430 [8.7%]	4,073 [6.4%]	367 [11.7%]
Colonoscopies, mean [SE]^a^	2.3 [2.0]	1.8 [1.3]	4.4 [3.0]	4.5 [3.1]	4.3 [3.0]	3.4 [2.5]
Disease Duration, mean [SE]	NA	NA	8.9 [7.8]	8.9 [7.6]	9.1 [8.1]	5.0 [6.7]

IBD, inflammatory bowel disease; UC, ulcerative colitis; CD, Crohn’s disease; IC, indeterminate colitis; SE, standard error; NA, not available.

^a^Per individual 2003–2019.

#### 2.3.2. Inpatient setting

Data on IBD disease activity were not available in the registered data. Performance of the colonoscopy in the inpatient [as opposed to outpatient] setting was used as a proxy for severe disease flare.

#### 2.3.3. Time period

The data were grouped into four time periods: 2003–2007, 2008–2011, 2012–2015, and 2016–2019. The reference period was 2003–2007.

#### 2.3.4. General anaesthesia

No data were available regarding which drugs were used for general anaesthesia; therefore, any general anaesthesia was compared with no anaesthesia. However, most endoscopy units in Sweden use propofol as an anaesthetic which has a short recovery time, and the individual can be discharged later the same day.

#### 2.3.5. Demographics

Age was categorised into four groups, from 0–30, 31–50, 51–70 and 71+. The youngest age group was set as the reference. For sex, men were set as the reference. The number of colonoscopies per individual over 2003–2019 is shown as a descriptive variable in Table 1.

#### 2.3.6. Procedures during colonoscopy

This included polypectomy, dilatation [JFA58], and biopsy [UJF35]. Polypectomy includes simple polypectomy [JFA15] and endoscopy mucosal resection [JFA85]. Information on the number or size of polyps is not included in the registers.

#### 2.3.7. Antithrombotic use

For antithrombotic medicine, the use of acetylsalicylic acid, other antiplatelet drugs, warfarin, and direct oral anticoagulants [DOACs], prescribed within the 3 months prior to the colonoscopy and registered in the Swedish Prescribed Drug Register, was used. Data regarding temporary stop of antithrombotic medicine in relation to the colonoscopies are not included in the register. However, most endoscopy units pause antithrombotic medicine before and during the colonoscopy, following recommendations from the Swedish Gastroenterology Society^17^ which are harmonised with the ESGE guidelines.^18^

### 2.4. Statistical analysis

Patient and procedure characteristics per colonoscopy are presented in Table 1. Bivariate logistic regression was used to compare the odds of bleeding and perforation between individuals with IBD and individuals without IBD and for the other risk factors, respectively. The multivariable analyses included bleeding and perforation, respectively, as outcome variables, and the following exposure variables: IBD status, setting, time period, general anaesthesia, sex, age, procedure during colonoscopy [polypectomy, dilatation and biopsy], and the use of anticoagulants. In addition, we performed moderation analyses to test if the odds of adverse events in patients with and without IBD differed between inpatient and outpatient settings. In the moderation analyses, an interaction between IBD status and setting was added to the multivariable analyses. The analyses were clustered on patient identity, as some patients underwent several endoscopies during the study period. Also, in Supplementary Table 1 we present crude rates as percentages to simplify interpretation and application to clinical practice. A *p*-value <0.05 was considered statistically significant. All analyses were performed using Stata 17 [StataCorp, College Station, TX].

## 3. Results

During the period 2003–2019, 969 532 colonoscopies were performed in 661 080 adult individuals. Of these, 164 012 colonoscopies were performed on individuals with an IBD diagnosis, of which 59% were performed in patients with UC, 39% in patients with CD, and 2% in patients with IC, see Table 1. The mean disease duration in patients with IBD was 8.9 years, and IBD patients underwent significantly more colonoscopies compared with patients without IBD [Table 1]. For further patient and procedure characteristics on the study population, see Table 1.

### 3.1. Bleeding

The bleeding rate for all colonoscopies was 0.19%. The odds of bleeding were significantly lower in individuals with IBD compared with individuals without IBD (crude odds ratio [OR] = 0.42, 95% confidence interval [CI]: 0.34–0.50, *p* <0.001, adjusted OR [AdjOR] = 0.66, p <0.001]. The odds of bleeding were significantly lower in UC compared with individuals without IBD [AdjOR = 0.62, *p *= 0.001], and CD compared with individuals without IBD [AdjOR = 0.69, *p *= 0.013]. The odds of bleeding were significantly higher in men, and increased with polypectomy, increasing age, and with antithrombotic use. Anaesthesia, dilatation, and biopsy were not associated with bleeding; see Table 2 for adjusted ORs. Crude ORs are presented in Supplementary Table 1.

**Table 2.** Risk factors for bleeding and perforation in colonoscopy, analysed with multivariable logistic regression using Swedish colonoscopy registry data from 2003 to 2019 [*n *= 661 080 patients, 969 532 colonoscopies].

	Bleeding	*p*	Perforation	*p*
	OR^a^ [95% CI]		OR^a^ [95% CI]	
IBD	0.66 [0.54–0.81]	< 0.001	0.79 [0.63–0.98]	0.033
Inpatient setting	4.87 [4.40–5.39]	< 0.001	4.37 [3.83–4.99]	<0.001
Period				
2003–2007	1 [Reference]	1 [Reference]	1 [Reference]	1 [Reference]
2008–2011	1.07 [0.91–1.27]	0.407	1.04 [0.86–1.25]	0.675
2012–2015	1.20 [1.02–1.41]	0.025	0.85 [0.70–1.02]	0.086
2016–2019	1.20 [1.02–1–40]	0.027	0.85 [0.70–1.03]	0.093
General anaesthesia	1.26 [0.87–1.82]	0.216	2.02 [1.42–2.87]	<0.001
Women	0.69 [0.62–0.76]	< 0.001	1.14 [1.003–1.29]	0.044
Age				
0–30	1 [Reference]	1 [Reference]	1 [Reference]	1 [Reference]
31–50	1.44 [1.02–2.04]	0.039	1.05 [0.69–1.60]	0.805
51–70	1.77 [1.28–2.45]	0.001	2.05 [1.42–2.98]	<0.001
71 +	1.74 [1.25–2.43]	0.001	2.85 [1.96–4.16]	<0.001
Biopsy	0.97 [0.88–1.07]	0.509	0.97 [0.85–1.10]	0.631
Polypectomy	3.54 [3.20–3.91]	< 0.001	2.14 [1.84–2.50]	<0.001
Dilatation	1.63 [0.73–3.63]	0.234	8.49 [5.34–13.50]	<0.001
Antithrombotic use	1.58 [1.40–1.77]	< 0.001	0.79 [0.68–0.93]	0.004

OR, odds ratio; CI, confidence interval; IBD, inflammatory bowel disease.

^a^Odds ratios [OR] adjusted for all variables included in the table.

#### 3.1.1. Outpatient vs inpatient setting

The odds of bleeding were higher for colonoscopies performed in inpatient settings compared with outpatient settings [AdjOR = 4.87, *p* = <0.001], Table 2. Setting moderated the association between IBD status and bleeding, so that patients with IBD had increased odds for bleeding in inpatient setting compared with patients without IBD [OR for interaction between setting and IBD status = 1.44, *p *= 0.031].

#### 3.1.2. Time period

The use of antithrombotic medicine and frequency of polypectomy increased from 2003–2007 to 2016–2019, see Table 3. The adjusted odds of bleeding were increased in the 2016–2019 period as compared with the 2003–2007 period [AdjOR = 1.20, *p* = 0.027].

**Table 3.** Rate of bleeding and perforation, the percentage of antithrombotic use, and polypectomy stratified by time period.

	2003–2007	2008–2011	2012–2015	2016–2019	Total
Bleeding					
No [*n*]	172 532	223 331	275 696	296 134	967 693
Yes [*n*]	245	383	545	666	1 839
%	0.14	0.17	0.20	0.22	0.19
Perforation					
No [*n*]	172 575	223 443	275 968	296 485	968 471
Yes [*n*]	202	271	273	314	1,060
%	0.12	0.12	0.10	0.11	0.11
Antithrombotic use^a^ [%]	9.98	19.11	19.35	21.21	18.19
Polypectomy [%]	6.98	8.89	12.16	20.52	13.04

^a^Taking one or more antithrombotic medicine at the time of colonoscopy.

### 3.2. Perforation

The perforation rate for all colonoscopies was 0.11%. The odds of perforation were significantly lower in IBD patients compared with individuals without IBD [crude OR = 0.57, 95% CI: 0.47–0.70, *p* <0.001, adjOR = 0.79, *p* = 0.033]. The odds of perforation were significantly lower in UC compared with individuals without IBD [adjOR = 0.61, p = 0.005], whereas no difference was found between CD and individuals without IBD [adjOR = 1.05, *p* = 0.752]. The odds of perforation were significantly higher in women and with polypectomy, dilatation, increasing age, and general anaesthesia, whereas antithrombotic use and biopsy were not associated with perforation risk. See Table 2 for adjusted ORs; crude ORs and crude rate are presented in Supplementary Table 1.

#### 3.2.1. Outpatient vs inpatient setting

The odds of perforation were higher for colonoscopies performed in inpatient settings compared with outpatient settings [adjOR = 4.37, *p* <0.001; Table 2]. Setting did not moderate the association between IBD status and perforation [OR for interaction 1.13, *p* = 0.568].

#### 3.2.2. Time period

There was no change in adjusted odds for perforation over time from 2003–2007 to 2016–2019 [adjOR = 0.85, *p* = 0.093]; see also Table 3.

## 4. Discussion

In this nationwide register study, IBD status was not associated with a higher rate of adverse events after colonoscopy. However, setting, time period, and procedures were associated with increased bleeding and perforation in colonoscopy. Inpatient IBD colonoscopies had higher odds of bleeding compared with colonoscopies in IBD patients in the outpatient setting. General anaesthesia was associated with a 2-fold increase in the odds of perforation. Women had lower odds of bleeding, but higher odds of perforation compared with men. Older age and polypectomy were associated with more frequent bleeding and perforation. The use of antithrombotic medicine was associated with higher odds of bleeding but not perforation. Dilatation was associated with increased odds of perforation but not bleeding.

The lower odds for bleeding and perforation in patients with IBD could be interpreted as indicating that IBD is protective against complications associated with colonoscopy. However, the lower risk may be due to residual confounding by indication. There is a qualitative difference in patients who undergo colonoscopy due to IBD compared with other indications such as bleeding or CRC surveillance. Non-IBD individuals are older, more often need polypectomy, and are more frequently treated with antithrombotic medicine. Although adjustment for these covariates was performed, it is likely that residual confounding variables that affect the outcome still exist. Overall, these data would suggest that IBD is not associated with higher risk of bleeding or perforation after a single colonoscopy. However over the study period, individuals with an IBD diagnosis underwent twice as many colonoscopies as individuals without IBD, increasing their cumulative lifetime risk of adverse events.

Previous studies have reported conflicting results regarding the likelihood of adverse events in IBD colonoscopies, and there is a lack of population-based studies. In a large cohort study including 612 190 colonoscopies [33 732 IBD colonoscopies] Navaneethan *et al.* found an increased risk of perforation during colonoscopy in IBD patients compared with non-IBD controls,^11^ whereas Ferreira *et al*. in a single-centre study found that the risk of adverse events after each endoscopy in IBD patients was comparable to the risk in the general population, although patients with IBD had an elevated lifetime risk of adverse events after colonoscopies.^13^ In a case-control study, Mukewar *et al.* reported a significantly higher rate of perforation in IBD patients compared with non-IBD patients [18.91 per 10 000 and 2.50 per 10 000 procedures, respectively, *p* = 0 001].^12^ Buisson *et al*. found no difference in perforation rate between IBD patients and controls in a large single-cent rerretrospective cohort study. However of 16 patients with perforation in the control group, most had other risk factors for perforation, such as colon cancer, dilatation procedures, or polypectomy.^14^

IBD disease activity is a factor that may be hypothesised to affect risk of complications after colonoscopy. Unfortunately, disease activity is not captured in the available data. Instead, colonoscopy performed in the inpatient setting was used as a proxy for active disease. In IBD patients undergoing colonoscopy in the inpatient setting compared with the outpatient setting, the odds of bleeding were almost seven times higher and the odds of perforation were almost five times higher. There are clear limitations to the extent to which inpatient status reflects IBD disease activity, and patients in this category may have been inpatients for other reasons, such as requirement for inpatient bowel cleansing or other conditions requiring inpatient care. Future studies linking endoscopic scores, histology, and colonoscopy outcomes might be able to address this question.

General anaesthesia was associated with a 2-fold increased likelihood of perforation. It may be hypothesised that this is due to the absence of feedback or pain signalling from the patient. Moreover, the lack of positional change might increase technical difficulty of the colonoscopy. Another possible explanation for the increase in adverse events associated with general anaesthesia is due to confounding by indication. For example, a stricture in the colon might cause more pain during colonoscopy and therefore prompt general anaesthesia, but the same stricture might also increase the likelihood of perforation. This finding of higher odds of perforation when general anaesthesia is employed is in keeping with previous reports. Both Cooper in the USA^19^ and in Adeyemo *et al.* in Spain^20^ demonstrated an increased risk of perforation during general anaesthesia. However, Bielawska *et al*. found no increase in perforation risk.^21^

The odds of bleeding increased from 2003–2007 to 2016–2019. Some of the increase may be explained by an increase in antithrombotic use as well as an increase in polypectomy frequency [Table 3]. During the study period, development in techniques/methods that replace surgery, such as endoscopy mucosal resection and endoscopic submucosal dissection, have made it possible to remove endoscopically larger polyps, which might also contribute to this increase.^22,23^ However, there was no increase in the odds of perforation over the 17-year study period, which might be considered counterintuitive when considering the increase in the removal of large polyps over the same period. On the other hand, these results might also reflect the increased training and skill among therapeutic endoscopists.

Other results of this study are mostly in line with previous studies, including the findings that polypectomy is associated with increased likelihood of bleeding and perforation,^2,4^ that the rates of bleeding and perforation increase with age,^4,24^ that antithrombotic medicine is associated with an increased risk of bleeding,^25,26^ and that dilatation is associated with an increased risk of perforation.^27^

The strength of this study is the nationwide, population-based design including almost one million colonoscopies. The population-based nature makes it less prone to selection bias compared with studies that employ patient selection, for example including patients entering a colorectal cancer screening programme or patients treated at tertiary referral centre.

The main limitation of this study, in common with other register-based studies, is that the analyses are limited to the data available in the registers. In the current study, we did not have information on disease activity and instead had to use a proxy for that in the form of inpatient setting. Other data that are not available and could affect the outcome are: if and at what time antithrombotic medicine was discontinued; if another antithrombotic medicine temporally replaced it; the completion rate of the colonoscopies; and bowel preparation. Another possible source of bias is in the rare case of an individual who has an adverse event in an outpatient setting and then gets admitted to a hospital; this would be registered as an inpatient colonoscopy. Finally, our definition of a bleeding or perforation, by using the codes T81.0 and T81.2 up to 30 days after a colonoscopy, might capture gastrointestinal [GI] bleeding caused by procedures other than colonoscopy. But we think this is rare, and it does not systematically affect one group more than another. As such, it should not skew the overall message of these data.

In conclusion, bleeding and perforation were rare and were not more common in colonoscopies in individuals with IBD compared with those without IBD. However, IBD inpatients had marked increased odds for adverse events, suggesting that disease activity may be a significant risk factor and that extra care is warranted in patients with IBD when they undergo colonoscopy as an inpatient. Several common procedures during colonoscopy increased the risk of adverse events in general. Notably, general anaesthesia during colonoscopy was associated with a greater risk of perforation.

## Supplementary Material

jjad114_suppl_Supplementary_Table_S1

## Data Availability

The data underlying this article cannot be shared publicly due to the privacy of individuals who participated in the study. The data will be shared on reasonable request, including an ethical permission that is approved, to the corresponding author.
