# Supplementary material for: Inflammatory Bowel Disease Is not Linked to a Higher Rate of Adverse Events in Colonoscopy—a Nationwide Population-based Study in Sweden
Source: J Crohns Colitis. 2023 Jul 4;17(12):1962–7. doi: 10.1093/ecco-jcc/jjad114 (PMC10798860; doi:10.1093/ecco-jcc/jjad114)
Supplement: jjad114_suppl_Supplementary_Table_S1 [file jjad114_suppl_supplementary_table_s1.docx]

Supplementary Table 1

Risk factors for bleeding and perforation in colonoscopy, analyzed with bivariate logistic regression and also presented as crude rate. From the Swedish colonoscopy registry data from 2003 to 2019, (n=661,080 patients, 969,532 colonoscopies).

|  | **Bleeding** |  |  | **Perforation** |  |  |
| --- | --- | --- | --- | --- | --- | --- |
|  | **OR (95% CI)** | **p** | **Crude rate** | **OR (95% CI)** | **p** | **Crude rate** |
| **Non-IBD** | 1 (Reference) |  | 0.21% | 1 (Reference) |  | 0.12% |
| **IBD** | 0.42 (0.34-0.50) | < 0.001 | 0.09% | 0.57 (0.47-0.70) | < 0.001 | 0.07% |
| **Ulcerative Colitis** | 0.38 (0.30-0.49) | < 0.001 | 0.08% | 0.37 (0.26-0.51) | < 0.001 | 0.04% |
| **Crohn’s Disease** | 0.45 (0.34-0.60) | < 0.001 | 0.09% | 0.87 (0.68-1.13) | = 0.307 | 0.10% |
| **Indeterminate Colitis** | 0.75 (0.23-2.46) | = 0.644 | 0.17% | 0.81 (0.26-2.52) | = 0.719 | 0.10% |
| **Inpatient setting** | 4.89 (4.46-5.36) | < 0.001 | 0.64% | 4.80 (4.25-5.43) | < 0.001 | 0.37% |
| **Period** |  |  |  |  |  |  |
| **2003-2007** | 1 (Reference) |  | 0.14% | 1 (Reference) |  | 0.12% |
| **2008-2011** | 1.21 (1.02-1.43) | = 0.029 | 0.17% | 1.04 (0.86-1.25) | = 0.707 | 0.12% |
| **2012-2015** | 1.39 (1.19-1.63) | < 0.001 | 0.20% | 0.85 (0.70-1.02) | = 0.075 | 0.10% |
| **2016-2019** | 1.58 (1.36-1.85) | < 0.001 | 0.22% | 0.90 (0.75-1.09) | = 0.282 | 0.11% |
| **General Anesthesia** | 0.97 (0.67-1.39) | = 0.859 | 0.18% | 1.70 (1.20-2.41) | = 0.003 | 0.18% |
| **Men** | 1 (Reference) |  | 0.24% | 1 (Reference) |  | 0.10% |
| **Women** | 0.62 (0.56-0.68) | < 0.001 | 0.15% | 1.12 (0.99-1.27) | = 0.070 | 0.12% |
| **Age** |  |  |  |  |  |  |
| **0-30** | 1 (Reference) |  | 0.06% | 1 (Reference) |  | 0.04% |
| **31-50** | 1.58 (1.12-2.23) | = 0.010 | 0.10% | 1.06 (0.69-1.61) | = 0.79880 | 0.05% |
| **51-70** | 2.89 (2.09-3.98) | < 0.001 | 0.18% | 2.29 (1.57-3.32) | < 0.001 | 0.10% |
| **71 +** | 4.55 (3.30-6.26) | < 0.001 | 0.29% | 4.12 (2.84-5.98) | < 0.001 | 0.18% |
| **Biopsy** | 0.80 (0.73-0.88) | < 0.001 | 0.17% | 0.79 (0.70-0.90) | < 0.001 | 0.11% |
| **Polypectomy** | 3.69 (3.36-4.06) | < 0.001 | 0.52% | 2.05 (1.78-2.37) | < 0.001 | 0.20% |
| **Dilatation** | 0.91 (0.41-2.02) | = 0.821 | 0.17% | 5.93 (3.82-9.23) | < 0.001 | 0.63% |
| **Antithrombotic** | 2.53 (2.28-2.81) | < 0.001 | 0.37% | 1.31 (1.13-1.52) | < 0.001 | 0.14% |
